# Genetic insights into morphometric inflorescence traits of wheat

**DOI:** 10.1007/s00122-019-03305-4

**Published:** 2019-02-14

**Authors:** Gizaw M. Wolde, Corinna Trautewig, Martin Mascher, Thorsten Schnurbusch

**Affiliations:** 1 Leibniz Institute of Plant Genetics and Crop Plant Research (IPK), Corrensstr. 3 OT Gatersleben, 06466 Seeland, Germany; 2 Present Address: Department of Plant Sciences, University of California, Davis, USA; 3 Faculty of Natural Sciences III, Institute of Agricultural and Nutritional Sciences, Martin-Luther-University, Halle-Wittenberg, 06120 Halle, Germany

## Abstract

**Key message:**

**Modifying morphometric inflorescence traits is important for increasing grain yield in wheat**. **Mapping revealed nine QTL, including new QTL and a new allele for the q locus, controlling wheat spike morphometric traits**.

**Abstract:**

To identify loci controlling spike morphometric traits, namely spike length (SL), internode length (IL), node number per spike (NPS), and node density (ND), we studied 146 Recombinant Inbred Lines of tetraploid wheat (*Triticum turgidum* L.) derived from standard spike and spike-branching mutant parents. Phenotypic analyses of spike morphometric traits showed low genetic coefficients of variation, resulting in high heritabilities. The phenotypic correlation between NPS with growing degree days (GDD) suggested the importance of GDD in the determination of node number in wheat. The major effect QTL for GDD or heading date was mapped to chromosome 7BS carrying the flowering time gene, *Vrn3*-*B1*. Mapping also identified nine QTL controlling spike morphometric traits. Most of these loci controlled more than a single trait, suggesting a close genetic interrelationship among spike morphometric traits. For example, this study identified a new QTL, *QND*.*ipk*-*4AL*, controlling ND (up to 17.6% of the phenotypic variance), IL (up to 11% of the phenotypic variance), and SL (up to 20.8% of the phenotypic variance). Similarly, the major effect QTL for IL was mapped to the *q* locus. Sequencing of the *Q*/*q* gene further revealed a new *q* allele, *q*^*del*^-*5A*, in spike-branching accessions possessing a six base pair deletion close to the miR172 target site. The identification of *q*^*del*^-*5A* suggested that the spike-branching tetraploid wheats are double mutants for the spikelet meristem (SM) identity gene, i.e., *branched head*^*t*^ (*TtBH*^*t*^), and the *q* gene, which is believed to be involved in the SM indeterminacy complex in wheat.

**Electronic supplementary material:**

The online version of this article (10.1007/s00122-019-03305-4) contains supplementary material, which is available to authorized users.

## Introduction

Wheat (*Triticum* sp.) domestication took place in the Fertile Crescent some 10,000 years ago (Heun et al. 1997; Faris 2014). Since then, it has become one of the largest and most important food crops grown across the world (Shiferaw et al. 2013). Like in other cereal crops, the domestication syndrome: a suite of phenotypic traits differentiating domesticated crops from their wild ancestors (Doebley et al. 2006; Meyer et al. 2012), is also evident in wheat. These include non-brittle rachis, grain threshability, spike length, tillering, photoperiod, and vernalization requirements (Salamini et al. 2002; Kilian et al. 2009; Faris et al. 2014).

Among several factors that have contributed to the broad adaptability of wheat in different parts of the world is the variation in flowering time (Beales et al. 2007; Cockram et al. 2007; Campoli and von Korff 2014). The time to flowering in wheat is predominantly influenced by developmental and environmental factors that are generally controlled by three sets of genes: the vernalization (*Vrn*), photoperiod (*Ppd*), and earliness per se genes (Yan et al. 2003, 2004, 2006; Beales et al. 2007; Cockram et al. 2007; Kippes et al. 2015).

Accelerated flowering in wheat resulted in reduced plant height, tillering, and spikelet number (Worland et al. 1998; Shaw et al. 2013). Furthermore, spikelet and floret primordia initiation in wheat are sensitive to temperature and photoperiod (Rawson 1970; Rahman and Wilson 1977, 1978; Cottrell et al. 1982; Baker and Gallagher 1983a, b; Worland et al. 1998; Boden et al. 2015; Steinfort et al. 2017). The sub-phase duration during spike development also affects spikelet number, thereby affecting grain yield (Rawson 1970; Baker and Gallagher 1983b; Guo et al. 2018). Therefore, temperature, light conditions, and phase duration are key factors for the variation in the spike morphometric traits, i.e., spike length, internode length, node density, and spikelet number (Friend 1965; Rawson 1970, 1971; Pinthus and Millet 1978; Fischer 1985; Rawson and Richards 1993; Shaw et al. 2013; Steinfort et al. 2017). As wheat spikelets bear the grains and are the building blocks of the wheat spike, spike morphometric traits are essential agronomic traits in wheat (Kumar et al. 2007).

At least three loci were known to control spike length in hexaploid wheat. These are the *q*/*Q*, *C* or *compactum* (i.e., club wheat) and the *s*/*S* (i.e., the shot wheat characterized by short dense spike) (Salina et al. 2000; Johnson et al. 2008; Faris et al. 2014). Therefore, common hexaploid wheat is suggested to have the genotype *QcS* (Faris et al. 2014). Among these loci, only the gene underlying the *q* locus has been identified as one of the domestication genes controlling spike length and other spike-related traits (Simons et al. 2006; Debernardi et al. 2017; Greenwood et al. 2017). Since the *C* and *s* loci were reported to reside on chromosome 2D and 3D, respectively, it is not yet clear whether the homoeo-loci also exist in tetraploid wheat.

As it has been suggested, if wheat yield is genuinely sink-limited (Slafer and Savin 1994; Borras et al. 2004; Shearman et al. 2005; Miralles and Slafer 2007), then increasing the sink (spike) size and improving spikelet fertility are key for increasing the grain yield (Donald 1968; Foulkes et al. 2011). In this regard, three approaches can be suggested for increasing the spike size in wheat. The first approach is increasing spikelet number per spike (Boden et al. 2015; Dobrovolskaya et al. 2015; Poursarebani et al. 2015; Dixon et al. 2018b). The second approach is increasing the number of florets and/or grains and grain size per spikelet (Debernardi et al. 2017; Greenwood et al. 2017; Guo et al. 2017; Sakuma et al. 2019). The third approach is increasing spikelet as well as floret/grain number and grain size simultaneously. The key challenge is, however, whether spike and/or spikelet fertility are proportionately improved with the increased sink size without trade-offs among these traits. Therefore, understanding the genetic, developmental and physiological basis of wheat spike morphology is key not only for increasing spikelet number and arrangements but also for maximizing the fruiting or grain setting efficiency of the spikelets (Slafer et al. 2015).

Despite the importance of wheat spike morphology, a relatively small number of genes have been identified (Simons et al. 2006; Dobrovolskaya et al. 2015; Poursarebani et al. 2015; Avni et al. 2017; Debernardi et al. 2017; Greenwood et al. 2017; Dixon et al. 2018b).

In this study, we used 146 F7- and F8-derived Recombinant Inbred Lines (RILs) derived from a cross between Bellaroi (an elite durum wheat cultivar with spring growth habit) and TRI 19165 (a traditional tetraploid landrace with winter growth habit and spike-branching commonly known as ‘Miracle Wheat’) for mapping the spike morphometric traits, namely spike length (SL), internode length (IL), node number per spike (NPS), node density (ND). The phenotypic and genetic analysis of spike morphometric traits generally shows high genetic correlation and heritability. Mapping also identified several genomic regions, including a new *q* allele (henceforth, *q*^*del*^-*5A*) controlling more than one trait, suggesting strong genetic interrelationship of these traits contributing to unique developmental outcomes affecting the wheat spike morphometric traits. Moreover, this study also identified a previously unidentified locus on chromosome 4AL, *QND*.*ipk*-*4AL*, controlling SL, ND, and IL. The QTL controlling ND and SL on chromosome 2AL (*QND*.*ipk*-*2AL*) was mapped to the region harboring the *C* (*Compactum*) locus of hexaploid wheat, suggesting that *QND*.*ipk*-*2AL* is likely to be the homoeolog in tetraploid wheat.

## Materials and methods

### Development of the RILs

One hundred forty-six F7-derived RILs were developed through single-seed descent (SSD) from an F2 population derived from a cross between Bellaroi (an elite durum wheat variety with spring growth habit) and TRI 19165 (a ‘Miracle Wheat’ and winter type). Since the mapping population segregated for winter/spring growth habit, all RILs with winter growth habit were excluded from the mapping population by growing all the F2 plants without vernalization in the greenhouse. Those that were able to complete their life cycle and give grains without vernalization were all spring types (*n* = 146) and were used for this study. The RILs were evaluated under field conditions for two consecutive years in 2014 and 2015 growing seasons in three different environments (IPK14, IPK15, and HAL15) in Germany. In 2014, the F7-derived RILs were evaluated in Gatersleben (IPK14), 51.49° N and 11.16° E, Germany. In the following season, i.e., 2015, the F8-derived RILs were evaluated in two environments: Gatersleben (IPK15) and Halle/Saale (HAL15), Germany. All field evaluations were conducted on 3.75 m^2^ plots in a Randomized Complete Block Design (RCBD) in two replications. Three hundred grains per m^2^ were sown in rows spaced at 20 cm. Plants in all locations were fertilized according to the soil conditions in each environment. Similarly, herbicides and fungicides were also applied in order to control weeds and fungal infestations.

### Phenotyping

Heading date was recorded when 50% of the spike appears from the flag leaf sheath in 50% of the plants following Zadoks scale (Zadoks 1985). Heading date was converted to growing degree days (GDD) (Miller et al. 2001) by summing the average daily maximum and minimum temperatures. Spike length, excluding the awns, was recorded from five to ten randomly sampled plants from the middle of each plot. Node number per spike was recorded from 10 to 15 spikes sampled from the middle row of each plot from all environments. Average spike internode length was calculated from spike length and node number per spike. Node density (average node number per cm of the spike) was computed from node number and spike length of the main culm spike by dividing node number by spike length. Basic statistical analysis, such as analysis of variance (ANOVA) and correlation analysis, was performed using Genstat 17 and SPSS 20 (IBM 2011; Payne et al. 2014). Narrow sense heritability was determined using the general formula: *h*^2^ = *σ*^2^*G*/(*σ*^2^*G* + *σ*^2^GEI + *σ*^2^*e*) (Nyquist 1991; Singh et al. 1993), where *σ*^2^G is genotypic variance due to additive effects, *σ*^2^GEI is variance component due to genotype-by-environment interaction, and *σ*^2^*e* is an error variance.

### DNA extraction and genotyping

Genomic DNA from all F7-derived RILs was isolated following the modified CTAB method as described by Doyle (1990) and used for mapping. The final concentration was measured and samples were used for CAPS marker analysis as well as for genotyping-by-sequencing (GBS) library preparation following two-enzyme genotyping-by-sequencing approach (Poland et al. 2012; Wendler et al. 2014).

### Marker generation from F7-RILs

DNA markers from RILs were generated through genotyping-by-sequencing (GBS) following the two-enzyme approach (Poland et al. 2012). Adapters were trimmed from reads with cutadapt version 1.8.dev0 (Martin 2011). Trimmed reads were mapped to the chromosome-shotgun assemblies of bread wheat cultivar Chinese Spring (The International Wheat Genome Sequencing Consortium (IWGSC) 2014) with BWA mem version 0.7.12 (Li 2013), converted to BAM format with SAMtools (Li et al. 2009) and sorted with Novosort (Novocraft Technologies Sdn Bhd, Malaysia, http://www.novocraft.com/). Multi-sample variant calling was performed with SAMtools version 0.1.19 (Li 2011). The command “mpileup” was used with the parameters “-C50 –DV”. The resultant VCF file was filtered with an AWK script provided as Text S3 by Mascher et al. (Mascher et al. 2013). Only bi-allelic SNPs were used. Homozygous genotype calls were set to missing if their coverage was below 1 or their genotype quality was below 3. Heterozygous genotype calls were set to missing if their coverage below 4 or their genotype quality was below 10. An SNP was discarded (i) if its quality score was below 40, (ii) its heterozygosity was above 20%, (iii) its minor allele frequency was below 10%, or (iv) had more than 66% missing data. Genotype calls were filtered and converted into genotype matrix with an AWK script available as Text S3 of Mascher et al. (2013) (Mascher et al. 2013). Chromosomal locations and genetic positions were taken from population sequence (POPSEQ) data (Chapman et al. 2015).

### Construction of the linkage map

The genetic linkage map was constructed using Joinmap 4.0 (Stam 1993). All distorted markers were removed based on goodness-of-fit using a Chi-squared test. Regression and maximum likelihood mapping algorithms were used for the linkage construction. Linkage groups were determined using Haldane’s mapping function. The maximum distance of 50 cM was used to determine linkage between two markers. The maps were drawn using Map chart version 2.3 (Voorrips 2002).

### QTL mapping

Phenotypic data from all 146 RILs and all environments (IPK14, IPK15, and HAL15) were used for QTL mapping using Genstat 17. A step size of 10 cM, minimum cofactor proximity of 50 cM, a minimum separation of selected QTL of 30 cM, and genome-wide significance level (alpha) of 0.05 were used for QTL analysis. Based on the mixed-model approach, the whole genome was scanned first using simple interval mapping (SIM) approach. Then, based on the SIM result, cofactors were selected for composite interval mapping (CIM). The final QTL model was selected using the backward selection on the selected cofactors, where QTL boundaries (lower and upper), QTL effect, and phenotypic variance explained (PVE) by QTL were determined.

### Development of CAPS marker

The diagnostic CAPS marker for the *Q*/*q* gene was developed based on SNP T3142C, located in the miR172 target site (Debernardi et al. 2017). The T3142C substitution differentiates the ancestral *q* allele from the derived, domesticated *Q* allele. Genome-specific primers PJG14 and PJG18 were used to amplify 230 bP fragments spanning T3142C (Greenwood et al. 2017). *Hinf I*, New England Biolabs^®^, USA, was used for restriction digestion analysis following manufacturer’s protocol. The *Hinf I* restriction site has been eliminated from the *Q*-*5A* allele due to the T3142C substitution. Thus, *Hinf I* digests only the amplicon from the ancestral allele, i.e., *q*-*5A* into two fragments, i.e., 158 and 72 bp (see Supplementary Figure. 1).

### Sequencing and sequence analysis

The complete *Q*/*q* gene (3229 bp) was amplified from genomic DNA of 44 spike-branching tetraploid wheat mutants (see Supplementary Table 2) using genome-specific primers (see Supplementary Table 1) and highly specific polymerase for amplification (HotStarTaq DNA Polymerase from QIAGEN). The amplicons were Sanger-sequenced from both sides using ten overlapping primer pairs of the *Q*/*q* gene. Sequences were assembled using Geneious version 11 (Kearse et al. 2012).

## Results

### Phenotypic correlation and heritability of spike morphometric traits

**Fig. 1:**
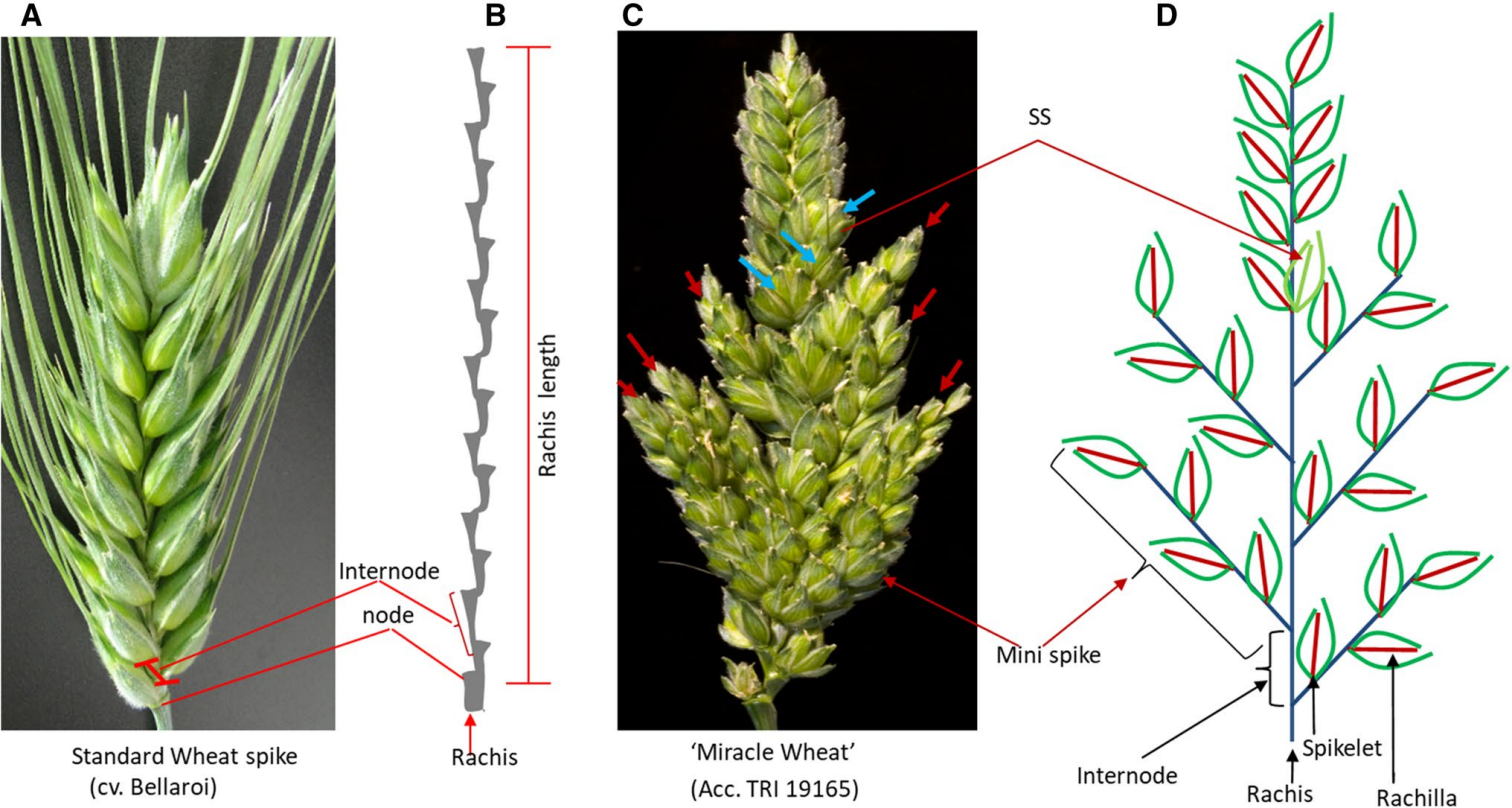
Spike morphological differences between parent Bellaroi (**A**) and TRI 19165 (**C**). For clarity, awns were removed from TRI 19165. Spike from Bellaroi shows a canonical or unbranched spike, where each node or spikelet is arranged in a distichous order along the axis (**B**). Red arrows in C show the mini-spike-like structures arising from the nodes of the bottom half of the rachis of TRI 19165, whereas the blue arrows show supernumerary spikelets (SS) sharing the same rachis node. (**D**) Schematic sketch of spike-branching wheat shown in **C** (color figure online)

The mapping population used in this study was developed from parents with contrasting spike morphology (Fig. 1). Pairwise correlations among spike morphometric traits: SL, NPS, IL, ND, and HD (GDD), are shown in Table 1. SL was positively correlated with NPS, HD, and IL but negatively correlated with the ND. ND showed a negative correlation with SL and IL, suggesting a trade-off between SL or IL with the spikelet density. NPS, which is the total number of nodes per spike, was also positively correlated with ND and HD but showed a negative correlation with IL. The effect of HD on SL and NPS was likely to be a pleiotropic effect. Previous studies have also demonstrated that accelerated flowering in wheat shortens the duration of the vegetative phase, including inflorescence primordia development (Worland et al. 1998; Miralles and Richards 2000; Shaw et al. 2013), thereby affecting the size and number of organs to be produced. Estimates of the phenotypic (*σ*^2^P), genotypic (*σ*^2^G), genotype-by-environment interaction (*σ*^2^GEI), and error variances (*σ*^2^*e*) for SL, NPS, ND, HD, and IL are also shown in Table 1. The *σ*^2^G for all the traits were higher than that of the *σ*^2^GEI, suggesting that the measured phenotypic variance was indeed controlled by the genetic factors underlying each trait. This conclusion was further supported by the low genetic coefficients of variation (GCV) and high heritabilities (h^2^) of the traits (Table 1).

**Table 1:** The phenotypic and genetic variances; Pearson’s correlation and heritability of spike morphometric traits

Trait	Variances, means, and *h*^2^							Pearson’s correlation				
	*σ*^2^P	*σ*^2^G	*σ*^2^GEI	*σ* ^2^ *e*	Mean	GCV (%)	*h* ^2^	SL	NPS	ND	HD (GDD)	IL
SL	0.98	0.82	0.08	0.08	7.14	12.67	84	1				
NPS	4.93	4.07	0.37	0.49	17.57	11.48	83	0.48**	1			
ND	0.14	0.11	0.01	0.02	2.50	13.00	78	− 0.60**	0.40**	1		
HD (GDD)	8715	7583	922	209	909	9.6	87	0.26**	0.58**	0.23**	1	
IL	0.004	0.003	0.00023	0.0007	0.41	13.23	76	0.61**	− 0.40**	− 0.98**	− 0.23**	1

σ^2^P phenotypic variance, *σ*^*2*^*G* genotypic variance, *σ*^*2*^*GEI* variance from the genotype × environment interaction, *σ*^2^*e* error variance, *GCV* genetic coefficients of variation, *h*^*2*^ narrow sense heritability, *SL* spike length, *NPS* node number per spike, *ND* node density, *HD* Heading date, *IL* internode length. The correlations are significant at *p* = 0.01 (**). (−) indicates negative correlation

### Identification of loci controlling spike morphometric traits

**Fig. 2:**
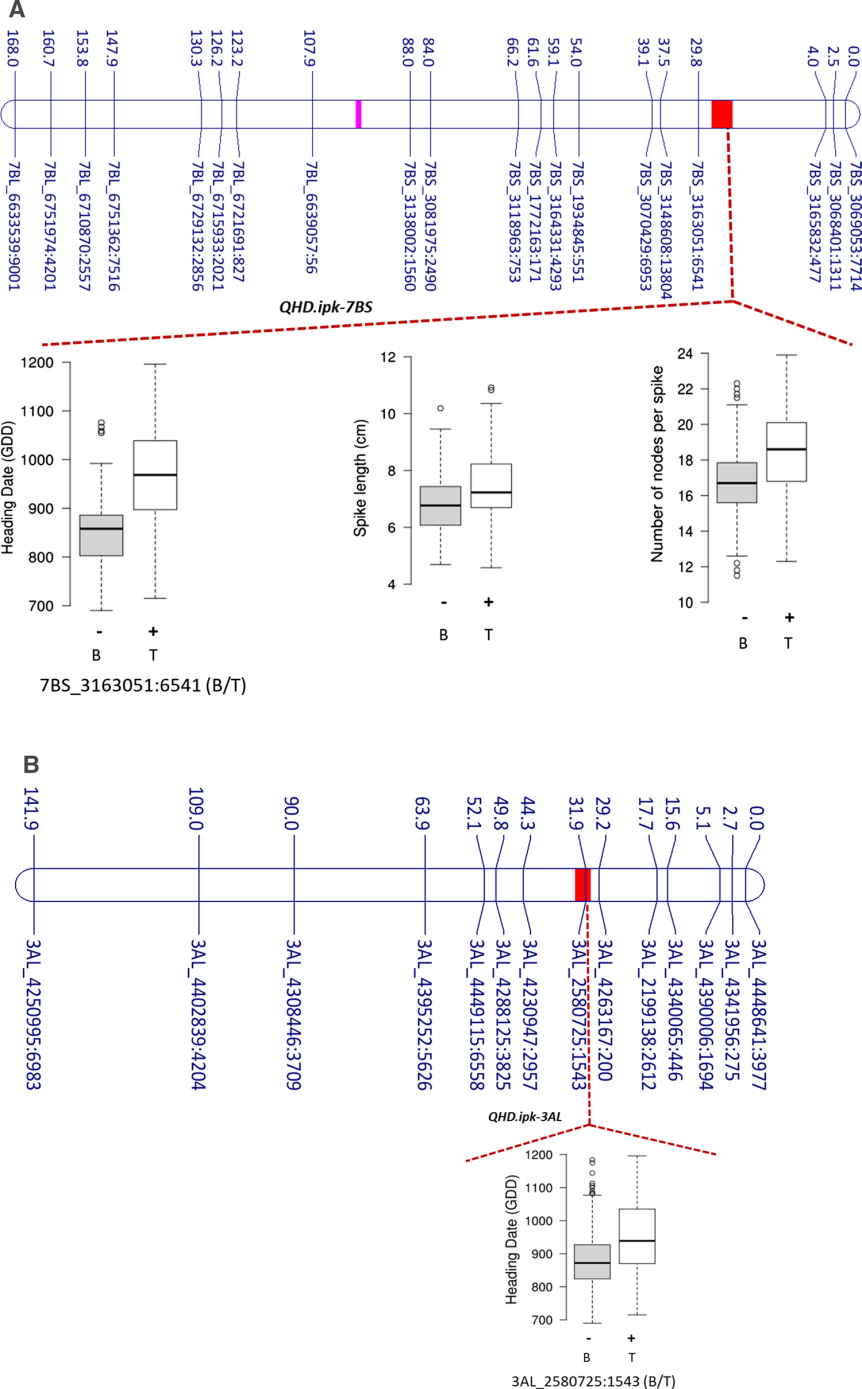

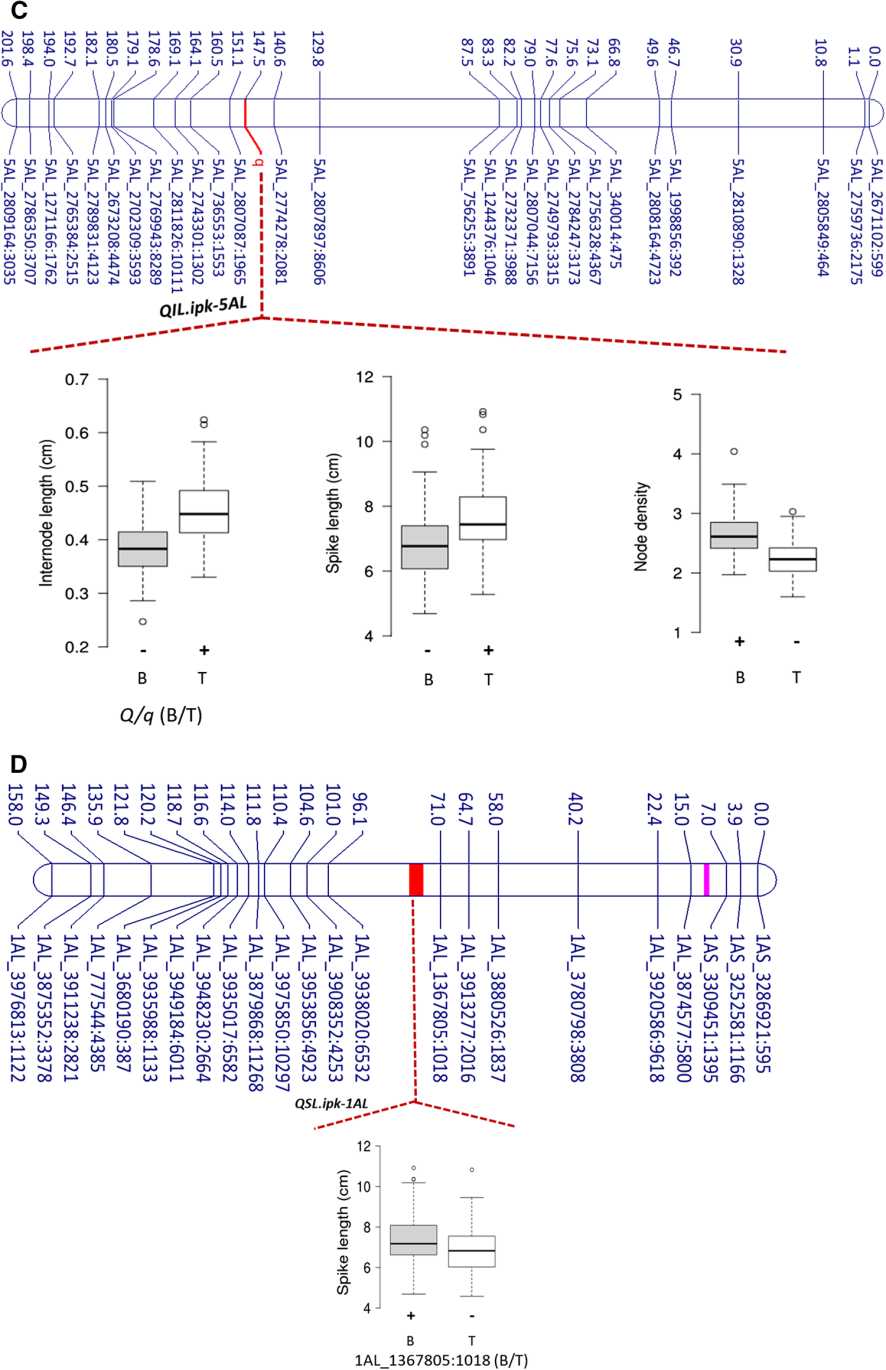

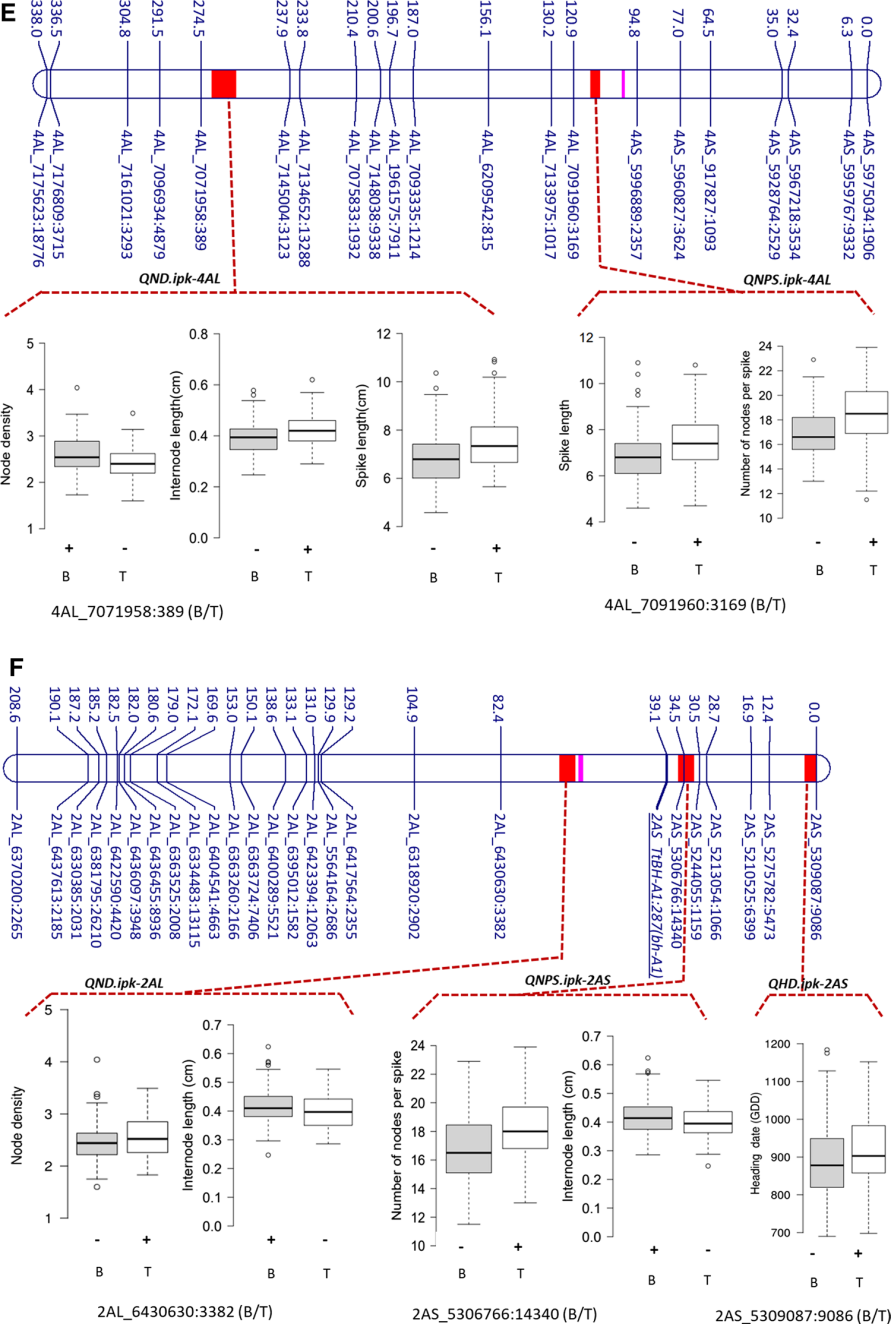
Genetic maps of chromosomes showing QTL for spike length (SL), node number per spike (NPS), node density (ND), heading date (HD), and internode length (IL). Effect of the QTL is shown as box plots computed after grouping the RILs into two sets based on the closest marker flanking each QTL. Grouping was made based on the closest marker for each QTL (shown below the box plot). (+) shows the phenotype of the RILs carrying the QTL, and (−) shows the phenotype of the RILs without the QTL. B, allele from Bellaroi; T, allele from TRI 19165. ‘Miracle Wheat’ allele or the *bh*^*t*^-*A1* allele on chromosome 2A (39.1 cM) is underlined. Pink dash shows the putative centromeric position per chromosome

Mapping identified several loci controlling spike morphometric traits. All the QTL, their chromosomal locations, QTL-by-environment additive effects (QTLxE AEs), and the phenotypic variance explained (PVE) by each of these QTL are shown in Table 2. Three QTL, i.e., *QHD*.*ipk*-*7BS*, *QHD*.*ipk*-*2AS*, and *QHD*.*ipk*-*3AL*, were detected for HD or GDD (Table 2, and Fig. 2A, B, F). The major effect QTL was located on chromosome 7BS (i.e., *QHD*.*ipk*-*7BS*). Interestingly, wheat chromosome 7BS carries the wheat flowering time gene *VRN3*-*B1* or *FT*-*B1* (Yan et al. 2006), suggesting that the underlying gene for *QHD*-*ipk*-*7BS* may indeed be *FT*-*B1*. *QHD*.*ipk*-*7BS* consistently appeared in all environments as a major effect QTL controlling about 59% of phenotypic variation for heading date. Similarly, wheat chromosome 2AS is known to carry the *PHOTOPERIOD RESPONSE* locus *Ppd*-*A1* (Beales et al. 2007), suggesting that the underlying locus for *QHD*.*ipk*-*2AS* is most likely *Ppd*-*A1*. The winter wheat accession TRI 19165 contributed the high-value alleles (HVAs; i.e., late flowering) for all the three-heading time QTL. Besides controlling heading date, *QHD*.*ipk*-*7BS* was also colocated with QTL for SL and NPS in all environments (Table 2, and Fig. 2A). The effect is likely to be pleiotropic due to delayed flowering (Shaw et al. 2013; Dixon et al. 2018a). This result was also supported by significant positive phenotypic correlations of HD with SL (0.26; *p *= 0.01) and NPS (0.58; *p *= 0.01; Table 1).

**Table 2:** QTL for spike length (SL), node number per spike (NPS), node density (ND), heading date (HD), and internode length (IL)

*QTL*	Genetic position (cM)	Trait	− log *p* value	Environment	QTLXE AEs	PVE (%)	HVA
*QHD*.*ipk*-*7BS*	25.83	HD	31.15	HAL15	77.9	49.8	TRI 19165
(*Vrn3*-*B1*)				IPK14	69.5	58.6	TRI 19165
				IPK15	53.2	48.5	TRI 19165
		NPS	8.8	HAL15	1.038	23.7	TRI 19165
				IPK14	1.038	21.1	TRI 19165
				IPK15	1.038	23.5	TRI 19165
		SL	8.4	HAL15	0.379	15.8	TRI 19165
				IPK14	0.211	6.8	TRI 19165
				IPK15	0.426	14.7	TRI 19165
*QHD*.*ipk*-*2AS*	0	HD	5	HAL15	16.06	2.1	TRI 19165
(*Ppd*-*A1*)				IPK14	16.06	3.1	TRI 19165
				IPK15	16.06	4.4	TRI 19165
*QHD*.*ipk*-*3AL*	31.9	HD	3.86	HAL15	27.24	6.1	TRI 19165
				IPK14	23.40	6.6	TRI 19165
				IPK15	17.67	5.4	TRI 19165
*QIL*.*ipk*-*5AL*	147.5	IL	35.9	HAL15	0.039	45.8	TRI 19165
(*q*)				IPK14	0.039	49.6	TRI 19165
				IPK15	0.039	38.9	TRI 19165
		SL	10.4	HAL15	0.45	22.2	TRI 19165
				IPK14	0.37	20.9	TRI 19165
				IPK15	0.54	23.1	TRI 19165
		ND	27	HAL15	0.23	40.9	Bellaroi
				IPK14	0.23	38	Bellaroi
				IPK15	0.23	43.3	Bellaroi
*QND*.*ipk*-*4AL*	265	ND	7	HAL15	0.145	16.7	Bellaroi
				IPK14	0.145	15.5	Bellaroi
				IPK15	0.145	17.6	Bellaroi
		IL	5.4	HAL15	0.018	10.1	TRI 19165
				IPK14	0.018	11	TRI 19165
				IPK15	0.018	8.6	TRI 19165
		SL	7.1	HAL15	0.369	15	TRI 19165
				IPK14	0.369	20.8	TRI 19165
				IPK15	0.369	11	TRI 19165
*QND*.*ipk*-*2AL*	65	ND	3.27	HAL15	0.185	27	TRI 19165
				IPK14	0.185	25	TRI 19165
				IPK15	0.185	28.5	TRI 19165
		IL	2.7	HAL15	0.018	9.7	Bellaroi
				IPK14	0.018	10.5	Bellaroi
				IPK15	0.018	8.2	Bellaroi
*QNPS*.*ipk*-*2AS*	34.5	NPS	8.4	HAL15	0.81	14.4	TRI 19165
				IPK14	0.81	12.8	TRI 19165
				IPK15	0.81	14.3	TRI 19165
		IL	3	HAL15	0.012	4.4	Bellaroi
				IPK14	0.012	4.8	Bellaroi
				IPK15	0.012	3.8	Bellaroi
*QNPS*.*ipk*-*4AL*	112	NPS	5.9	HAL15	0.863	16.4	TRI 19165
				IPK14	0.863	14.6	TRI 19165
				IPK15	0.863	16.3	TRI 19165
*QSL*.*ipk*-*1AL*	79.4	SL	5.3	HAL15	0.282	8.8	Bellaroi
				IPK14	0.282	12.2	Bellaroi
				IPK15	0.282	6.4	Bellaroi

*QTLXE AEs* QTL-by-environment interaction additive effects, *PVE* phenotypic variance explained, *HVA* high-value allele

The other spike morphometric trait studied in the current mapping population is spike internode length (IL). IL, also known as rachis IL, was defined as the length between two successive nodes on the rachis, which is the central axis of the spike (Fig. 1B). In total, four QTL were identified controlling IL (Table 2, Fig. 2C, E, F). By controlling up to 50% of the phenotypic variance, the QTL on chromosome 5AL, *QIL*.*ipk*-*5AL*, was the major effect QTL. *QIL*.*ipk*-*5AL* also affected SL and spikelet density (ND). Interestingly, chromosome 5AL carries the *q* locus, which is one of the domestication-related wheat genes controlling the square-head spike shape and other several spike-related traits (Simons et al. 2006; Debernardi et al. 2017; Greenwood et al. 2017). This result suggested that the underlying phenotypic variation for IL, SL, and ND at *QIL*.*ipk*-*5AL* was likely to be controlled by allelic variation at the *q* locus. A *Q*/*q* gene derived CAPS marker analysis further revealed allelic variation at the *q* locus between Bellaroi and TRI 19165. Expectedly, the commercial variety Bellaroi carried the modern *Q* allele (Supplementary Figure 1). To our surprise, TRI 19165 carried the ancestral *q* allele, which lacks the mutation within the miRNA172 binding site (Debernardi et al. 2017). Linkage analysis also placed the *q*-derived CAPS marker at cM position 147.5 on chr 5AL (Fig. 2C). Re-mapping of IL, ND, and SL after including the *q*-derived CAPS marker further suggested linkage of *q* with the phenotypes. Therefore, it is highly likely that allelic variation at the *q* locus, *QIL*.*ipk*-*5AL*, is responsible for the phenotypic variation in IL, ND, and SL.

Apart from *QIL*.*ipk*-*5AL*, two other loci from chromosome 4AL (*QND*.*ipk*-*4AL*) and 2AL (*QND*.*ipk*-*2AL*) also controlled ND, IL, and SL. By controlling ND as a primary effect, *QND*.*ipk*-*4AL* also controlled IL, thereby affecting SL as well. As far as we know, *QND*.*ipk*-*4AL* is a new QTL having strong effects on spike morphometric traits in tetraploid wheat. Given the strong negative correlation between ND and IL (− 0.98, *p *= 0.01; Table 1), TRI 19165 contributed the HVA for increased IL, thereby increasing SL. The corresponding allele from Bellaroi increased ND, maximizing spikelet number per unit length of the rachis. Similarly, *QND*.*ipk*-*2AL* was mapped to chromosome 2AL (Fig. 2F). TRI 19165 contributed the HVA for ND while the corresponding allele from Bellaroi increased the IL. Interestingly, Johnson et al. (2008) also mapped the *C* (*Compactum*) locus in hexaploid wheat on the long arm of chromosome 2D close to the centromeric region (Johnson et al. 2008), indicating that the *QND*.*ipk*-*2AL* is likely to be the homoeo-locus of *Compactum* in the tetraploid wheat, which was not reported before. Similarly, we cannot completely rule out the possibility that the underlying gene for *QND*.*ipk*-*2AL* could also be the wheat ortholog of barley *APELATA2* (*HvAP2*) gene which has been known to affect the density of grains along the rachis (Houston et al. 2013).

*QIL*.*ipk*-*2AS* controlling IL resides in a 4.6-cM interval distal to the *bh*^*t*^-*A1* locus (Poursarebani et al. 2015). Apart from controlling IL, *QIL*.*ipk*-*2AS* also had an effect on NPS (Table 2, Fig. 2F). The allele from Bellaroi increased IL, while the corresponding allele from TRI 19165 increased NPS, suggesting that *QIL*.*ipk*-*2AS* is either pleiotropic or closely linked to the ‘Miracle Wheat’ or the *bh*^*t*^-*A1* allele. Consistent with this, earlier studies have also mapped ND in the region harboring the wheat *frizzy panicle* (*WFZP*) gene which is the ortholog of *TtBH*^*t*^*1* in hexaploid wheat (Sourdille et al. 2000; Ma et al. 2007; Cui et al. 2012; Echeverry-Solarte et al. 2014; Dobrovolskaya et al. 2015; Echeverry-Solarte et al. 2015).

### Genetic interrelationships of spike morphometric traits are revealed by shared QTL

**Fig. 3:**
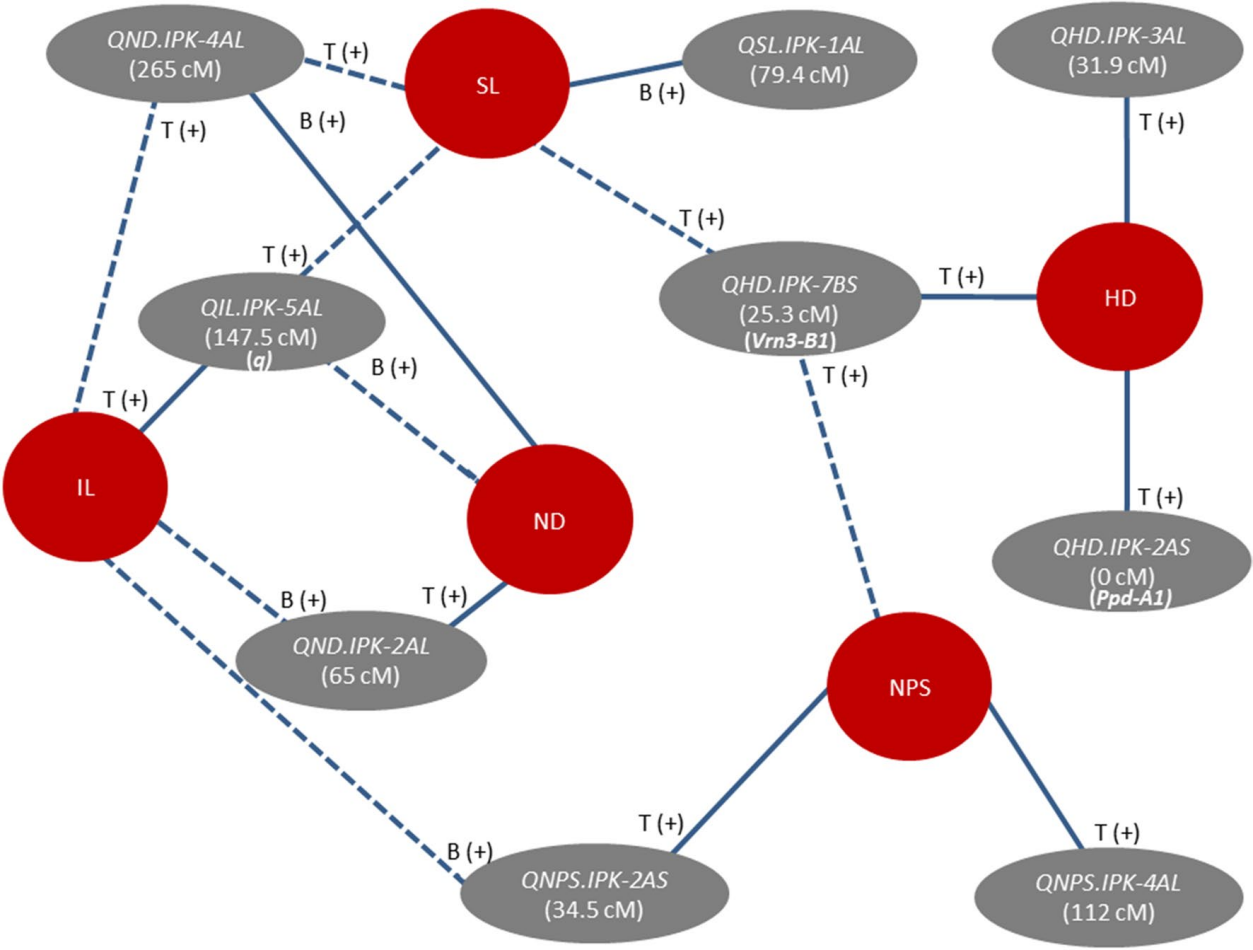
Genetic interrelationship of the spike morphometric traits as revealed by shared QTL. Solid lines connect the major effect QTL with the trait. Broken lines connect the extended effect of the QTL on the correlated traits. T(+) or B (+) indicates the source of the high-value allele (HVA) where T indicates allele from TRI 19165 and B indicates the corresponding allele from Bellaroi. *HD* heading date, *IL* internode length, *SL* spike length, *ND* node density, *NPS* node number per spike

Besides the phenotypic correlations among spike morphometric traits, shared QTL among traits suggested genetic interrelationships between spike morphometric traits (Fig. 3). Correlation analysis showed that SL and NPS were positively correlated with HD (Table 1). Although the effect of the heading date QTL on chr 7BS, *QHD*.*ipk*-*7BS*, on SL and NPS seemed to be a pleiotropic effect, it might be the cause for the genetic correlation between SL and NPS with HD. Similarly, IL was strongly positively correlated with SL and negatively correlated with ND. Likewise, *QIL*.*ipk*-*5AL*, which is a major effect QTL for IL, also colocated with SL as well as ND. *QND*.*ipk*-*4AL*, which is the major effect QTL for ND, also colocated with SL and IL (Table 2). The strong negative correlation between IL and ND was also clearly manifested by the reciprocal action of the parental alleles at *QIL*.*ipk*-*5AL* and *QND*.*ipk*-*4AL*, where alleles from Bellaroi increased ND, while the corresponding alleles from TRI 19165 increased the IL (Fig. 3). Taken together, these results suggest a tight interplay among common gene sets that control spike morphometric traits providing unique developmental outcomes during spike development.

### Sequence analysis from the spike-branching wheat mutants revealed a new *q* allele

**Fig. 4:**
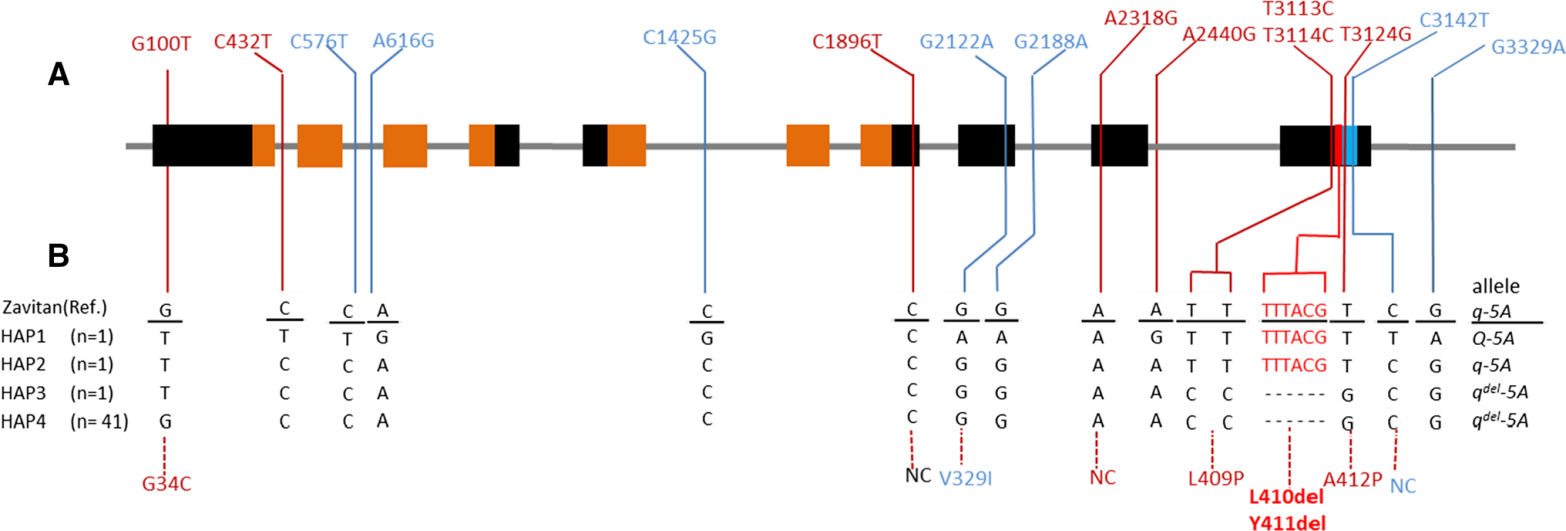
Structural variations at the *q* locus in the spike-branching ‘Miracle Wheat’ mutants. **A** The *q* gene model. The box indicates exons. The orange filled boxes indicate the coding regions for the two AP2 domains of the *q* protein. SNP positions are indicated at the top. The numbers indicate the position of each SNP from the start codon. Each SNP was designated by taking Zavitan as a reference sequence. SNPs in red fonts are identified in this study, whereas those with blue fonts are also previously reported including the T3142C substitution that differentiated the two alleles at the *q* locus (Simons et al. 2006; Debernardi et al. 2017). The red and blue boxes in exon 10 indicate the six bp deletion and miR172 binding site, respectively. **B** Haplotypes of the spike-branching mutants at the *q* locus. Amino acid changes due to the non-synonymous substitutions are indicated beneath the figure. L410del and Y411del are deleted amino acids due to the deleted nucleotides. NC, no change; HAP, haplotype. The sequences from each haplotype reported in this study are deposited in the GenBank database with accession numbers MK423900, MK423901, MK423902, and MK423903 (color figure online)

After discovering allelic variation at the *Q*/*q* locus between Bellaroi and TRI 19165, we sequenced this locus in 44 different spike-branching wheat mutants (Supplementary Table 2.). Forty-two out of the 44 spike-branching mutants carried a six bp deletion in exon 10, designated as *q*^*del*^-*5A* (Fig. 4 and Supplementary Figure 2). The six bp deletion resulted in the deletion of two concurrent amino acids (L410del and Y411del) close to the miR172 target site; however, deletion and miR172 binding site do not overlap. From the fifteen SNPs identified (Fig. 4), eight of them were identified in this study. From these eight SNPs, six SNPs, i.e., G100T, C1896T, A2318G, T3113C, T3114C, and T3124G, were identified in the protein coding sequences. Among these, four SNPs, namely G100T, T3113C, T3114C, and T3124G, were non-synonymous (Fig. 4). While the T3113C and T3114C occurred outside of the miR172 target site, T3124G was located within the 3′-end of the miR172 target site, which is generally considered to be a less conserved region for miRNAs binding as compared to the 5′-end, or the so-called seed region (Bartel 2009). From previous studies, it was known that the interaction between miR172 and modern *Q* allele was reduced due to the pivotal SNP (C3142T), resulting in high *Q* transcript levels and shorter IL in modern square-headed spikes (Debernardi et al. 2017). Carriers of the new *q*^*del*^-*5A* allele, however, rather show relaxed spike architecture (larger IL), implying that either transcript levels (less likely by T3124G) and/or amino acid substitutions (L409P, L410del, Y411del, A412P) might reduce or impair q^del^-5A protein function in ‘Miracle Wheats.’ Even if we have not yet established the exact effect of the mutation for the *q*^*del*^-*5A* allele, the increased IL or SL was likely to be the effect of the *q*^*del*^-*5A* allele (Fig. 2C). Taken together, these results unambiguously showed that most ‘Miracle Wheats’ carry two mutations in two independent genes: one for spike-branching (i.e., *bh*^*t*^-*A1* allele) and one for the speltoid-like or spear-shaped spike form (i.e., *q*^*del*^-*5A* allele).

## Discussion

### Wheat spike morphometric traits are phenotypically and genetically correlated with high heritabilities

Since its domestication, wheat has undergone important morphological changes in the inflorescence (Faris 2014). Although only a few genes were identified in this regard (Simons et al. 2006; Avni et al. 2017), the molecular understanding of the morphological changes of the inflorescence is not only important for the understanding of crop evolution and domestication, but also important for increasing grain yield through genetic manipulation of inflorescence architecture. Due to the complex genome structure of wheat (Mayer et al. 2014), its inflorescence architecture is controlled in a complex way. This often creates difficulties of visualizing the morphological changes caused by any recessive mutations due to the buffering effect of other functional gene(s) from the sub-genomes (Krasileva et al. 2017).

Wheat spike morphometric traits related to the domestication of the spike include SL, ND, NPS, and IL. Although several genetic loci were detected for these traits (Echeverry-Solarte et al. 2015; Zhai et al. 2016; Zhou et al. 2017), the *Q* gene is the only one that has been identified (Simons et al. 2006). Similarly, in the current population QTL for SL, ND, and IL were also mapped to the region of the *q* locus on 5AL (Fig. 2C). Interestingly, another important genomic region on 4AL, *QND*.*ipk*-*4AL*, similarly controls SL, ND, and IL. Most of the previous studies mapped SL QTL on 4AL in a region spanning from 75 to 93 cM (Jantasuriyarat et al. 2004; Kumar et al. 2007; Chu et al. 2008; Echeverry-Solarte et al. 2015). By controlling NPS and SL, *QNPS*.*ipk*-*4AL* was mapped at cM position 112 (Table 2, and Fig. 2E) and therefore is likely to be identical with previous studies. However, *QND*.*ipk*-*4AL* was mapped at cM position 265 (Table 2, and Fig. 2E) and hence is likely to be a new QTL.

As the wheat chromosome 4A is well characterized by two reciprocal translocations and two inversions, a portion of 4AL corresponds to 4DS (Mickelson-Young et al. 1995; Miftahudin et al. 2004; Dvorak et al. 2018). Likewise, the QTL reported by Cui et al. (2012) on 4DS controlling SL, NPS, and ND might correspond to *QND*.*ipk*-*4AL* (Cui et al. 2012). Recently, Dixon et al. (2018a, b) also reported the interaction between *TEOSINTE BRANCHED1*(*TB1*) and *FLOWERING LOCUS T1*, (i.e., *Vrn3*-*B1*) regulating inflorescence architecture in bread wheat (*T*. *aestivum* L.). Since *TB1* is located on the short arm of chromosome 4D (i.e., 4DS), we checked whether *TB*-*A1* could be the underlying gene for *QND*.*ipk*-*4AL*. *QND*.*ipk*-*4AL* was approximately mapped between 629,347,058 and 634,848,225 bp, while *TB*-*A1* was mapped between 582,841,008 and 582,839,894 bp, indicating that *TB*-*A1* is most likely not the candidate gene for *QND*.*ipk*-*4AL* but likely for *QNPS*.*ipk*-*4AL* which was flanked by marker 4AL_7091960:3169, mapped at position 544,598,274 bp. Therefore, the identification of genes underlying loci controlling spike morphometric traits, especially *QND*.*ipk*-*2AL*, *QND*.*ipk*-*4AL*, and *QNPS*.*ipk*-*4AL*, is essential for a better genetic and molecular understanding of spike morphogenesis in wheat.

### Rachis, spikelets, rachilla, and florets are the building blocks of wheat spike architecture

**Fig. 5:**
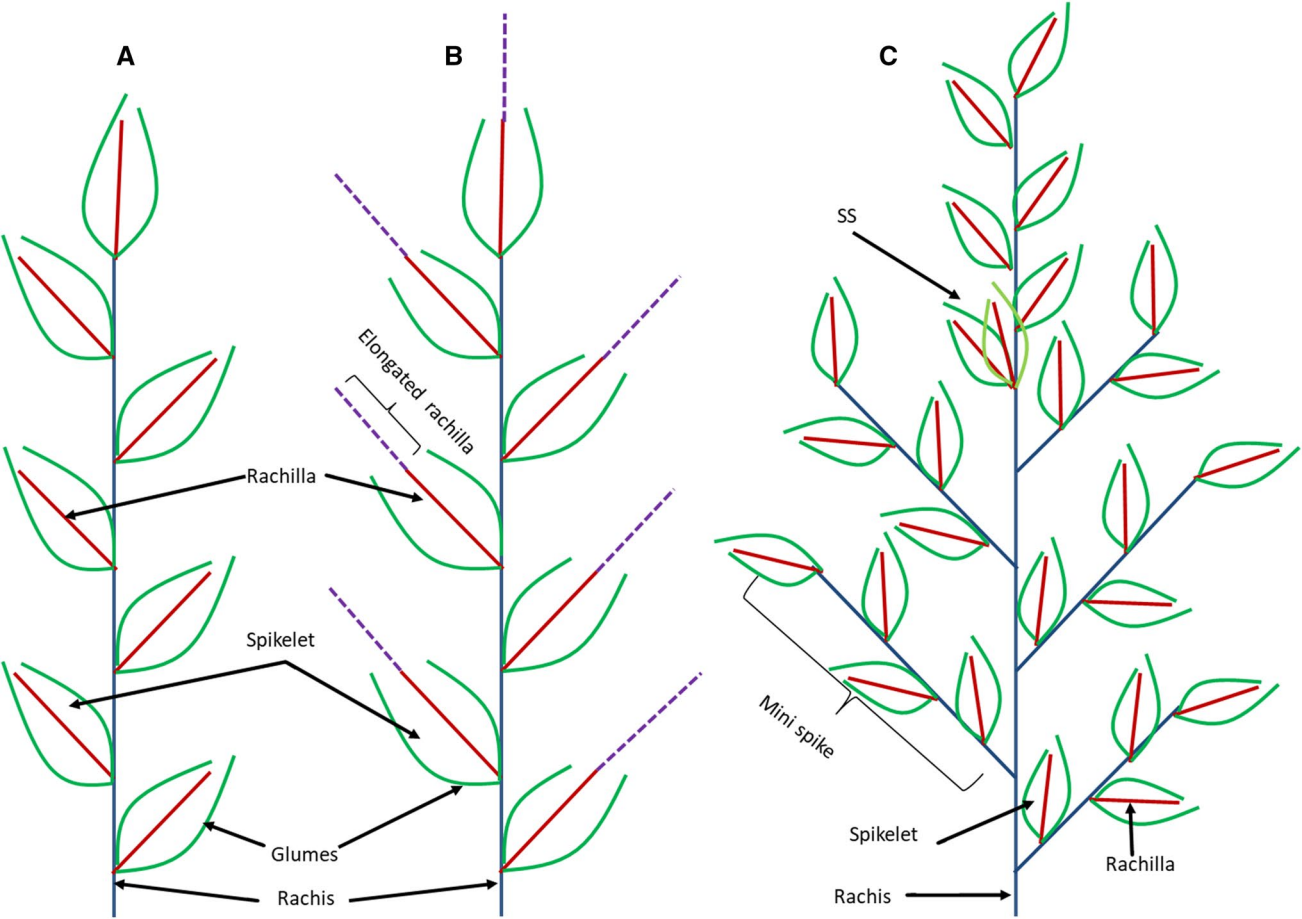
Wheat spike architecture. **A** The canonical or standard wheat spike where spikelets are arranged in a distichous order along the spike axis or the rachis. **B** The *Q* gene null mutants showing elongated rachilla (broken line) carrying more florets (not shown) as reported by Debernardi et al., (2017). **C** Spike-branching wheat mutant showing mini-spike arising from rachis node in place of the spikelets. *SS* supernumerary spikelet

Inflorescence architecture is determined by the activity of the inflorescence meristem (Tanaka et al. 2013). In wheat, the inflorescence meristem (IM) directly produces the spikelet meristems (SMs) on its flanks. The SMs then differentiate into glume primordia (GP) and floral meristems (FMs) (Barnard 1955; Kirby and Appleyard 1984). Due to the determinate meristematic activity of the IM, the wheat spike initiates a terminal spikelet, thereby terminating the initiation of new SMs from the IM (Bonnet 1967; McMaster 1997). However, unlike other cereal crops, the wheat SMs differentiate indeterminately, thereby initiating several floral meristems (FMs) on the flanks of the central spikelet axis, known as the rachilla (Fig. 5). Each FM finally differentiates into floral organs: lemma, palea, stamen, lodicules, and pistil. In standard or canonical wheat spike architecture, branching from the rachis node or the development of supernumerary spikelets (SS) is highly suppressed, while primary sessile spikelets are distichously attached to the central axis of the inflorescence, known as rachis (Fig. 5A).

Spike-branching and/or SS formation is prevented by TtBH^t^-A1/WFZP that provides SM identity to the axillary meristems (AxMs) of the IM (Dobrovolskaya et al. 2015; Poursarebani et al. 2015). Thus, spike-branching *TtBH*^*t*^-*A1* mutants rather re-initiate inflorescence-like meristems instead of directly forming SMs, implicating that these mutants have partially lost their SM identity. Interestingly, the newly developed inflorescence-like meristems also initiate new AxMs, which will finally acquire the identity of SMs, thereby producing complete sessile spikelets arranged distichously on a central axis that resembles the main rachis (Fig. 5C).

Recent studies have shown that the wheat *Q* gene is a suppressor of floret number through the suppression of rachilla development (Debernardi et al. 2017; Greenwood et al. 2017). This has been confirmed by *Q* gene mutants of wheat which are typically characterised by rachis (Simons et al. 2006) and rachilla extension with extra florets (Debernardi et al. 2017; Greenwood et al. 2017). Therefore, the Q protein is also involved in the SM indeterminacy complex, i.e., by regulating rachilla development and the morphogenesis of the rachis. Based on this, it is very likely that lower abundance of the q^del^-5A protein in ‘Miracle Wheats’ might also lead to a similar phenotype, i.e., extension of the rachilla with more florets. In fact, we found one such case in our RIL population, implying that ‘Miracle Wheats’ are indeed double mutants of SM identity and determinacy genes, i.e., *bh*^*t*^-*A1* and *q*^*del*^-*5A*, respectively.

As SM identity and determinacy are two interdependent processes during grass inflorescence development (Chuck et al. 2002; Laudencia-Chingcuanco and Hake 2002; Whipple 2017; Bommert and Whipple 2018); and the fact that SM identity and regular spikelet patterning/determinacy are not affected by the loss-of-function of the Q protein (Debernardi et al. 2017; Greenwood et al. 2017), it is likely that the TtBH^t^/WFZP and q proteins have rather a non-redundant function in wheat.

### Flowering time genes pleiotropically control spikelet number in wheat

Among the key developmental decisions plants have to make in their life cycle is whether and when to flower (Lin 2000). Such decisions, however, are dependent on the internal (genetic components) and external (environmental) factors including nutrients, temperature, day length, drought, salinity, exogenously applied hormones and chemicals (Cho et al. 2017). Furthermore, the time of flowering in plants is also accompanied by massive developmental reprogramming, resulting in the morphological, physiological, and biochemical changes (Poethig 1990). The most noticeable of all is the emergence of the inflorescence or reproductive structures. The fact that the current mapping population has been developed from contrasting parents, originating from distinct gene pools differing for flowering time (i.e., winter vs. spring), showed that early-heading RILs are characterized by a relatively short spike with less number of nodes (spikelets), while the late-heading RILs had relatively longer SL carrying more spikelets (Fig. 2A). This suggests the importance of phase duration, especially from spike initiation to the appearance of the terminal spikelet (Rawson 1970; Rahman and Wilson 1977; Guo et al. 2018) for increasing spikelet number in wheat. Likewise, accelerated flowering has been linked to reduced spikelet and tiller numbers in wheat (Worland et al. 1998; Shaw et al. 2013; Boden et al. 2015), suggesting that besides the benefits of flowering time genes for adjusting flowering time to certain climatic conditions and requirements, they are also important for the genetic modification of inflorescence architecture in wheat. Given the apparent effect of flowering time on inflorescence development and architecture (Guo et al. 2018), unraveling the molecular mechanisms of flowering may have a major impact for improving grain yield in wheat.

#### Author contribution statement

TS conceived and supervised the project. GMW conducted the experiments, collected, and analyzed the data. CT contributed in developing the RILs, isolation of DNA, and performed PCR and *q* gene sequencing. MM analyzed GBS data. GMW and TS wrote the manuscript. All authors read and approved the final version of the manuscript.

## Electronic supplementary material

Below is the link to the electronic supplementary material.

Supplementary material 1 (PDF 553 kb)
