# Supplementary material for: Genetic insights into morphometric inflorescence traits of wheat
Source: Theor Appl Genet. 2019 Feb 14;132(6):1661–76. doi: 10.1007/s00122-019-03305-4 (PMC6531419; doi:10.1007/s00122-019-03305-4)
Supplement: Supplementary file 1 — Supplementary material 1 (PDF 553 kb) [file 122_2019_3305_MOESM1_ESM.pdf]

Supplementary Table 1. List of primers

| Name | sequences | Purpose |
|---|---|---|
| PJG31 | GATGGTGCTGGATCTCAATGTGG * | Genomic amplification of *5A-q/Q* gene |
| G5A-R1 | GCGAGAGACCAGCCAGTAGT | |
| PJG14 | TCACTGCTGGTGCTGGTGC* | Genomic sequence spanning miRNA 172-Binding site |
| PJG 18 | AAGTAGAACCGGTGGTGGTCC * | |
| QA-R1 | GCTCACCCAAGTAGACCTGC | Sanger sequencing |
| QA-F1 | TCTGGTCAGCCCTCCTCTCGTCT | |
| QA-F2 | TGAGCGACTACGAGGAGGAT | |
| QA-F3 | TGTTCATGCTGTGGACGTTT | |
| QA-F4 | TTTGGGCTCACAAAACTTCC | |
| QA-R3 | CAGCGATATCAAAAGGCACA | |
| QA-R4 | ATCTAAAGCTGTGCCATGGG | |
| QA-F5 | TGGGTGTAGTGATGTGTCTG | |
| QA-F6 | TGCAGGTGCAGGTGCAGGA | |

* Taken from Greenwood et al., (2017), *Development* 144**,** 1959-1965

Supplementary Table 2. List of accesions used for sequencing the *q/Q* locus

| **Acc.** | ***Taxon*** | **Country** | **Spike** | **Allele** | **Haplotype** |
|---|---|---|---|---|---|
| CItr17442 | *Triticum turgidum spp. polonicum* | USA | normal | *Q-5A* | |
| PI624725 | *Triticum turgidum spp. durum* | Iran | normal | *Q-5A* | |
| PI624053 | *Triticum turgidum spp. durum* | Iran | normal | *Q-5A* | |
| TRI4045 | *Triticum turgidum L* | EUR | branched (weak) | *Q-5A* | HAP1 |
| PI626935 | *Triticum turgidum spp. durum* | Iran | normal | *q-5A* | |
| TRI9753 | *Triticum dicoccon Schrank* | IND | normal | *q-5A* | |
| PI278645 | *Triticum turgidum spp. turgidum* | UK | branched (weak) | *q-5A* | HAP2 |
| PI361757 | *Triticum turgidum spp. polonicum* | Denmark | normal | $q^{del}$*-5A* | |
| PI623936 | *Triticum turgidum spp. durum* | Iran | normal | $q^{del}$*-5A* | |
| TRI3261 | *Triticum turgidum L.* | ESP | Branched | $q^{del}$*-5A* | HAP3 |
| TRI28396 | | ITA | Branched | $q^{del}$*-5A* | HAP4 |
| TRI27966 | *Triticum turgidum L.* | | Branched | $q^{del}$*-5A* | HAP4 |
| TRI19165 | *Triticum turgidum L.* | | Branched | $q^{del}$*-5A* | HAP4 |
| TRI18959 | | FRA | Branched | $q^{del}$*-5A* | HAP4 |
| TRI9652 | *Triticum turgidum L.* | CSK | Branched | $q^{del}$*-5A* | HAP4 |
| TRI9628 | *Triticum turgidum L.* | IND | Branched | $q^{del}$*-5A* | HAP4 |
| TRI9548 | *Triticum turgidum L.* | ARM | Branched | $q^{del}$*-5A* | HAP4 |
| TRI5283 | *Triticum turgidum L.* | CHN | Branched | $q^{del}$*-5A* | HAP4 |
| TRI4653 | *Triticum turgidum L.* | AUS | Branched | $q^{del}$*-5A* | HAP4 |
| TRI4461 | *Triticum turgidum L.* | EUR | Branched | $q^{del}$*-5A* | HAP4 |
| TRI4448 | | EUR | Branched | $q^{del}$*-5A* | HAP4 |
| TRI4446 | *Triticum turgidum L.* | HUN | Branched | $q^{del}$*-5A* | HAP4 |
| TRI4341 | *Triticum turgidum L.* | EUR | Branched | $q^{del}$*-5A* | HAP4 |
| TRI4270 | *Triticum turgidum L.* | ITA | Branched | $q^{del}$*-5A* | HAP4 |
| TRI4082 | *Triticum turgidum L.* | PRT | Branched | $q^{del}$*-5A* | HAP4 |
| TRI3411 | *Triticum turgidum L.* | SU | Branched | $q^{del}$*-5A* | HAP4 |
| TRI3365 | *Triticum turgidum L.* | CHN | Branched | $q^{del}$*-5A* | HAP4 |
| TRI3241 | *Triticum turgidum L.* | CAN | Branched | $q^{del}$*-5A* | HAP4 |
| TRI1782 | *Triticum turgidum L* | GER | Branched | $q^{del}$*-5A* | HAP4 |
| TRI1781 | *Triticum turgidum L* | GER | Branched | $q^{del}$*-5A* | HAP4 |
| TRI984 | *Triticum turgidum L* | EUR | Branched | $q^{del}$*-5A* | HAP4 |
| PI628173 | *Triticum turgidum spp. turgidum* | Iran | branched | $q^{del}$*-5A* | HAP4 |
| PI624726 | *Triticum turgidum spp. turgidum* | Iran | branched | $q^{del}$*-5A* | HAP4 |
| PI624691 | *Triticum turgidum spp. turgidum* | Iran | branched | $q^{del}$*-5A* | HAP4 |
| PI624457 | *Triticum turgidum spp. turgidum* | Iran | branched | $q^{del}$*-5A* | HAP4 |
| PI624057 | *Triticum turgidum spp. turgidum* | Iran | branched (weak) | $q^{del}$*-5A* | HAP4 |
| TRI19292 | *Triticum turgidum L.* | Iran | Branched | $q^{del}$*-5A* | HAP4 |

| | | | | | |
|---|---|---|---|---|---|
| PI623956 | *Triticum turgidum spp. turgidum* | Iran | branched | $q^{del}$-*5A* | HAP4 |
| PI623928 | *Triticum turgidum spp. turgidum* | Iran | branched | $q^{del}$-*5A* | HAP4 |
| PI623927 | *Triticum turgidum spp. turgidum* | Iran | branched | $q^{del}$-*5A* | HAP4 |
| PI620973 | *Triticum turgidum spp. turgidum* | Iran | branched | $q^{del}$-*5A* | HAP4 |
| PI620972 | *Triticum turgidum spp. turgidum* | Iran | branched | $q^{del}$-*5A* | HAP4 |
| PI620944 | *Triticum turgidum spp. turgidum* | Iran | branched | $q^{del}$-*5A* | HAP4 |
| PI620943 | *Triticum turgidum spp. turgidum* | Iran | branched | $q^{del}$-*5A* | HAP4 |
| PI438971 | *Trticum turgidum L.* | KAZ | branched | $q^{del}$-*5A* | HAP4 |
| PI349056 | *Trticum turgidum L.* | ARM | branched | $q^{del}$-*5A* | HAP4 |
| PI345413 | *Triticum turgidum spp. turgidum* | Bosnia and Herzegovina | branched | $q^{del}$-*5A* | HAP4 |
| PI286075 | *Triticum turgidum spp. turgidum* | Poland | branched | $q^{del}$-*5A* | HAP4 |
| PI225309 | *Triticum turgidum spp. turgidum* | Iran | branched | $q^{del}$-*5A* | HAP4 |
| CITr13713 | *Trticum turgidum L.* | USA | branched | $q^{del}$-*5A* | HAP4 |
| CITr13712 | *Triticum turgidum spp. turgidum* | USA | branched | $q^{del}$-*5A* | HAP4 |

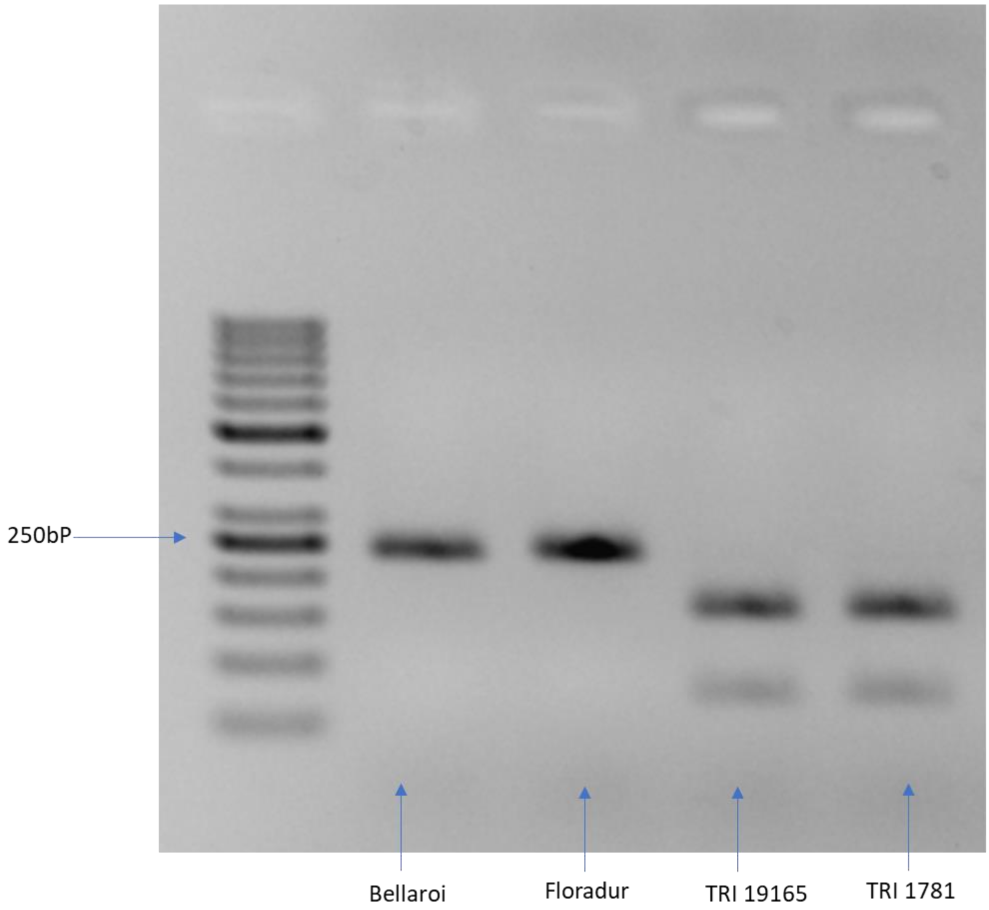


Supplementary Figure 1. The *Q* gene derived diagnostic CAPS marker used to differentiate the ancestral allele *q* from the modern allele *Q*. Bellaroi and Floradur are commercial durum wheat varieties carring the *Q* allele while TRI 19165 and TRI 1781 are land races with *q* allele.

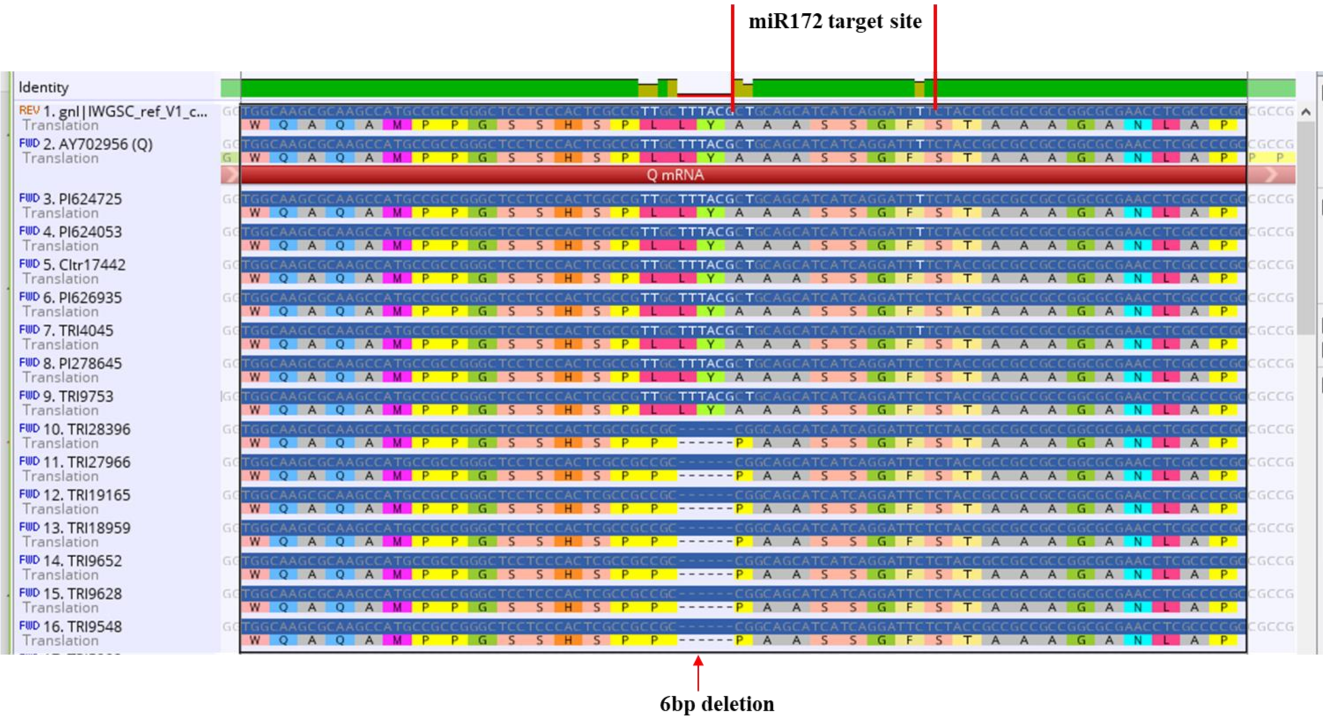
miR172 target site
Identity
REV 1. gnl|IWGSC_ref_V1_c...
FWD 2. AY702956 (Q)
Q mRNA
FWD 3. PI624725
FWD 4. PI624053
FWD 5. Cltr17442
FWD 6. PI626935
FWD 7. TRI4045
FWD 8. PI278645
FWD 9. TRI9753
FWD 10. TRI28396
FWD 11. TRI27966
FWD 12. TRI19165
FWD 13. TRI18959
FWD 14. TRI9652
FWD 15. TRI9628
FWD 16. TRI9548
6bp deletion

1/ 3

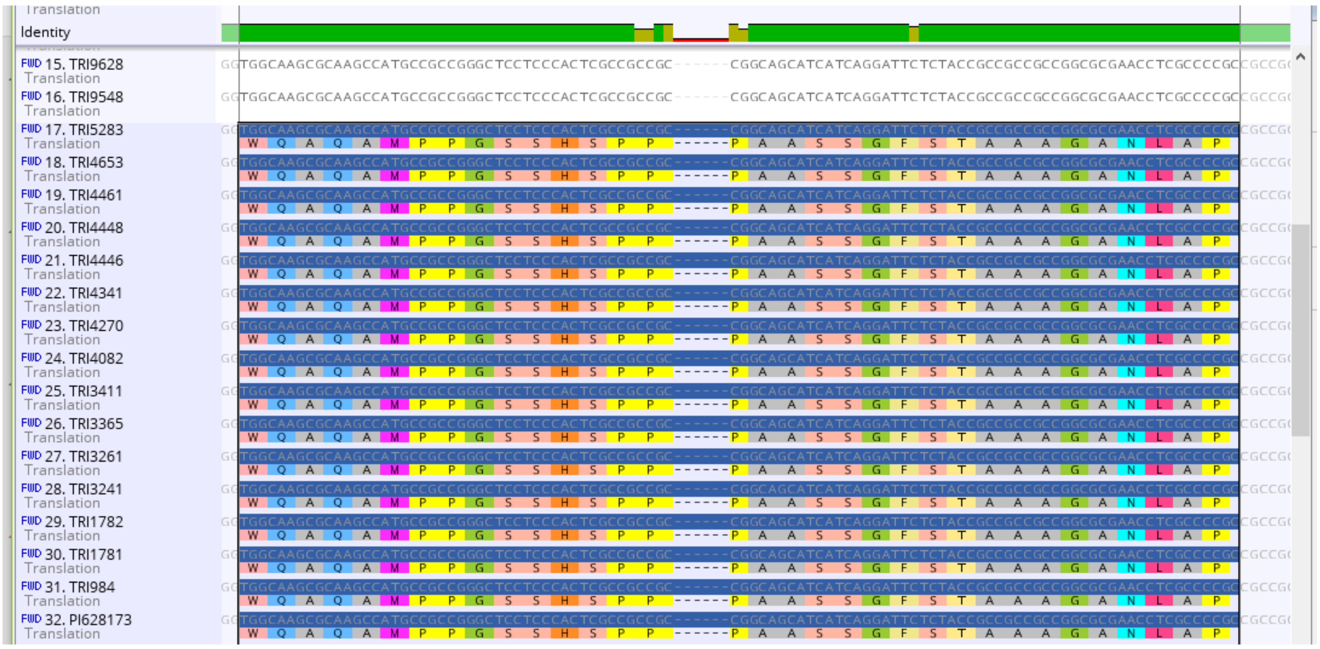
Identity
FWD 15. TRI9628
FWD 16. TRI9548
FWD 17. TRI5283
FWD 18. TRI4653
FWD 19. TRI4461
FWD 20. TRI4448
FWD 21. TRI4446
FWD 22. TRI4341
FWD 23. TRI4270
FWD 24. TRI4082
FWD 25. TRI3411
FWD 26. TRI3365
FWD 27. TRI3261
FWD 28. TRI3241
FWD 29. TRI1782
FWD 30. TRI1781
FWD 31. TRI984
FWD 32. PI628173

2/3

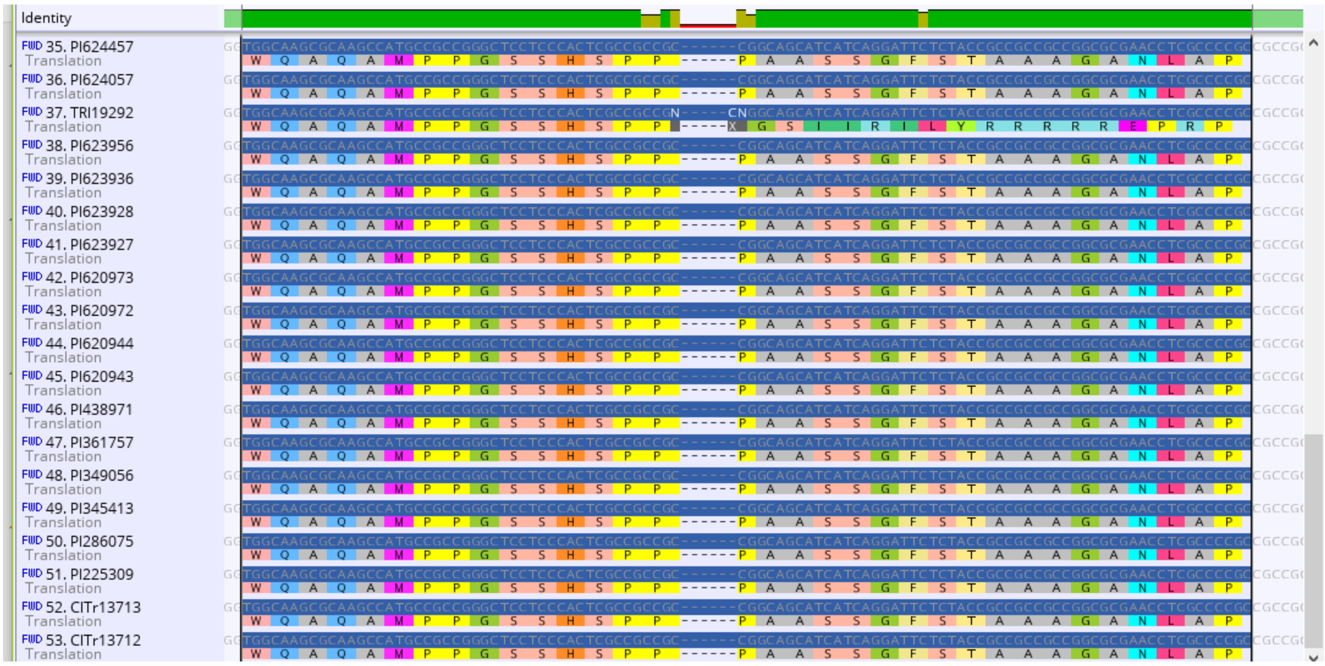

3/3

Supplementary Figure 2. Sequence alignment of the *q/Q* gene from spike-branching/non branching teraploid wheat accessions. Only region spanning the miR172 target site is shown here.
